# Pathogenesis of Oxidative Stress Biomarkers in Atrial Fibrillation: A Narrative Review

**DOI:** 10.19102/icrm.2026.17012

**Published:** 2026-01-15

**Authors:** Hira Naveed, Aqib Ishaque, Atif Nadeem, Aneeza Waris Hussain Rathore, Sadia Akhtar, Sara Haroon, Saira Rafaqat

**Affiliations:** 1 Department of Cardiology, Army Cardiac Hospital/CMH Lahore Medical College, Lahore, Pakistan; 2 Department of Zoology, Lahore College for Women University, Lahore, Pakistan; 3 Bachelor’s Science in Nursing, Aligarh College of Nursing, Lahore, Pakistan; 4 Department of Zoology (Molecular Physiology and Endocrinology), Lahore College for Women University, Lahore, Pakistan

**Keywords:** Atrial fibrillation, biomarkers, oxidative stress, pathogenesis

## Abstract

Atrial fibrillation (AF) is the most prevalent cardiac arrhythmia. Although its pathogenesis remains incompletely elucidated, accumulating evidence implicates oxidative stress (OS) as a key contributor to the development of the arrhythmogenic substrate. OS may facilitate atrial remodeling through modulation of calcium-handling proteins and ion channel function, potentially promoting the initiation and maintenance of AF. This article summarizes the role of OS biomarkers such as 8-hydroxydeoxyguanosine (8-OHdG), glutathione peroxidase (GPx), advanced glycation end-products (AGEs), superoxide dismutase (SOD), malondialdehyde (MDA), isoprostanes (IsoPs), derivatives of reactive oxidative metabolites that oxidize reduced glutathione (E_h_ GSH) and cysteine, and advanced oxidation protein products (AOPPs) in the pathogenesis of AF. Plasma 8-OHdG levels progressively increase with advancing low-voltage area stages, indicating a strong association between oxidative DNA damage and the severity of atrial fibrosis. Also, the reduction in GPx activity appears to contribute to arrhythmogenic electrochemical disturbances and oxidative lipid damage, independent of dyslipidemia. The receptor for the AGE axis plays a part in arrhythmogenic structural atrial remodeling. Patients who develop post-surgical AF demonstrate paradoxically elevated SOD activity, possibly reflecting a compensatory antioxidant response to heightened OS. As serum MDA levels were not linked to the development of postoperative AF (POAF), it is possible that lipid peroxidation is not the primary cause of POAF pathogenesis. Even when AF patients are receiving anticoagulant medication, elevated 8-isoprostane levels are linked to thromboembolic events, in part because of changes in the fibrin clot structure. Each 10% increase in E_h_ GSH was associated with a 40% increase in the risk of incident AF. Future research is required on AOPPs in AF pathogenesis. Future investigations should aim to identify and characterize novel OS markers and evaluate their potential therapeutic relevance in the prevention and management of AF.

## Introduction

Atrial fibrillation (AF) has emerged as a global epidemic of cardiovascular disease (CVD) and represents the most common clinically significant cardiac arrhythmia in the 21^st^ century.^1^ AF represents one of the most common cardiac arrhythmias, with an estimated lifetime risk affecting approximately one in three to five individuals after the age of 45 years. The number of people with AF increased significantly from 33.5 to 59 million globally between 2010 and 2019.^2^ AF is predicted to affect 6–12 million individuals in the United States by 2050 and 17.9 million in Europe by 2060. Significant morbidity and death, as well as substantial economic burden, are caused by AF, which is a key risk factor for ischemic stroke. Particularly, in nations with a middle sociodemographic index, AF incidence and prevalence have risen over the past 20 years and will do so for the next 30 years, making it one of the biggest epidemics and public health issues.^3^

Re-entry and/or fast focal ectopic firing can sustain AF. The “driver” is a common term for the mechanism that keeps AF going. A single localized re-entry circuit or local ectopic firing may provide an irregular atrial response to a quickly discharged, regularly firing driver, which would explain the erratic atrial discharge characteristic of AF. It is also possible that several functional re-entry circuits that change in time and place directly produce fibrillatory activity. The term “arrhythmogenic remodeling” describes any change in structure or function that encourages arrhythmias. In the majority of the acquired types of AF, remodeling is central. The fundamental processes that underlie AF have significant implications for AF-management protocols, such as those concerning rhythm regulation, rate regulation, and thromboembolism prevention.^4^

A biomarker is “a characteristic that is objectively measured and evaluated as an indicator of normal biological processes, pathogenic processes, or pharmacological responses to a therapeutic intervention,” according to the U.S. National Institutes of Health.^5^ The simplicity and cost of assessment, a biomarker’s performance qualities (such as sensitivity and specificity), and evidence for directing treatment and enhancing patient outcomes are all factors that define its clinical value.^6^ Biomarkers that closely match the disease’s pathophysiological process are the most promising. Oxidative stress (OS) is known to have a part in the pathogenesis of CVD.^7,8^ Nicotinamide adenine dinucleotide phosphate (NADPH) oxidase, mitochondria, xanthine oxidase, and uncoupled nitric oxide (NO) synthases are among the numerous sources of reactive oxygen species (ROS).^9^

OS biomarkers include molecules that are altered by interactions with ROS in the microenvironment and antioxidant system molecules that are altered in response to elevated redox stress. In vivo, excessive ROS can alter molecules such as proteins, carbohydrates, lipids (particularly phospholipids), and DNA. Some of these changes are known to directly affect the molecule’s function (eg, by inhibiting the activity of an enzyme), whereas others just indicate the level of OS present in the surrounding environment. It is acknowledged that one of the primary factors influencing the validity of the marker is the functional importance or causative effect of the oxidative alteration on cell, organ, and system function. An ROS biomarker’s clinical applicability is also influenced by the assay’s specificity, sensitivity, and reproducibility in measuring the modification; the biomarker’s stability under different storage conditions and specimen preparation procedures; and the ease of obtaining a suitable biological specimen.^10^

## Methods

**Figure 1:**
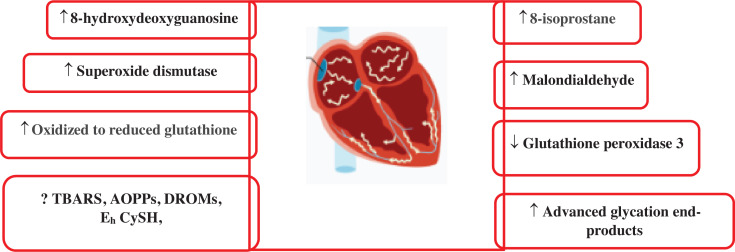
Circulatory levels of oxidative stress biomarkers in atrial fibrillation. Increased levels (↑), decreased levels (↓), and not determined yet (?) in atrial fibrillation, derivatives of reactive oxidative metabolites that oxidize reduced glutathione and cysteine, advanced oxidation protein products, and thiobarbituric acid reactive substances. *Abbreviations:* AF, atrial fibrillation; AOPPs, advanced oxidation protein products; DROMs, derivatives of reactive oxidative metabolites; E_h_ CySH, oxidized to reduced cysteine; E_h_ GSH, oxidized to reduced glutathione; TBARS, thiobarbituric acid reactive substances.

**Figure 2:**
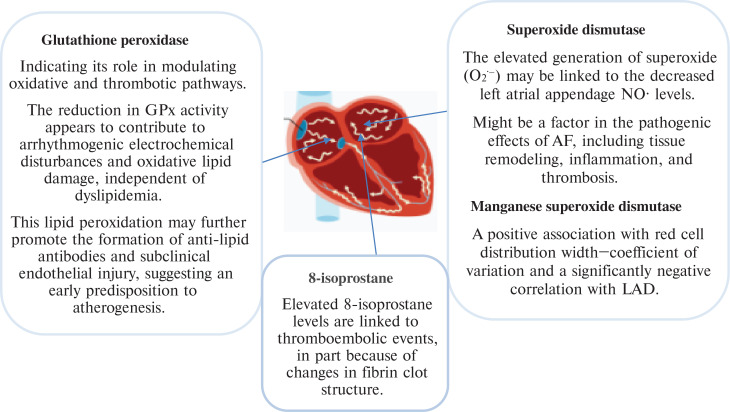
Overall pathogenesis of glutathione peroxidase, 8-isoprostane, and superoxides in atrial fibrillation. *Abbreviations:* GPx, glutathione peroxidase; LAD, left atrial diameter.

**Figure 3:**
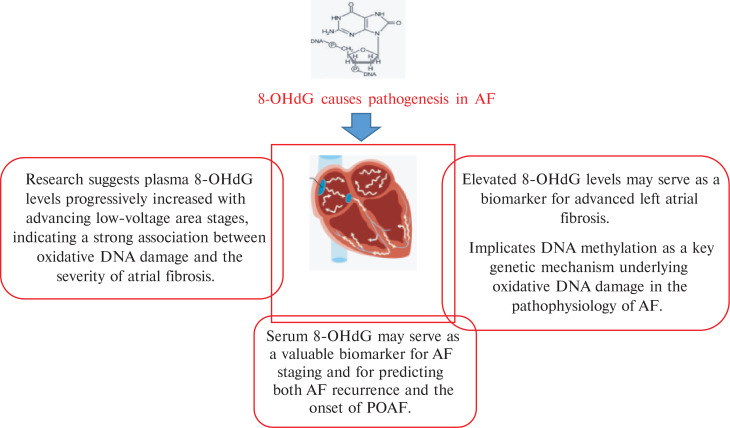
8-OHdG causes pathogenesis in AF. *Abbreviations:* AF, atrial fibrillation; 8-OHdG, 8-hydroxydeoxyguanosine; POAF, postoperative atrial fibrillation.

**Figure 4:**
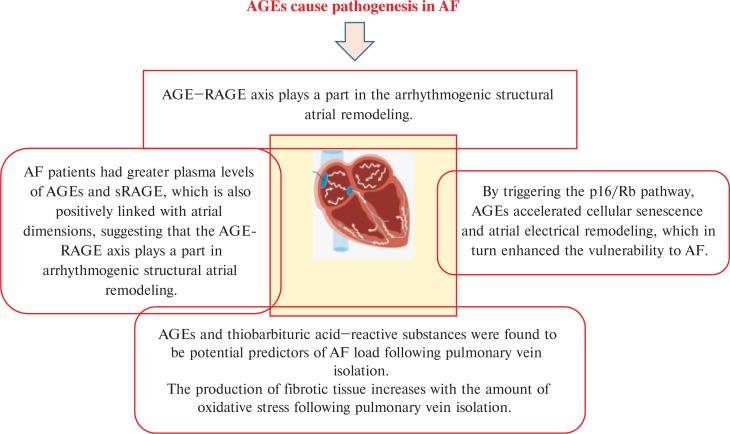
AGEs cause pathogenesis in AF. *Abbreviations:* AGEs, advanced glycation end-products; AF, atrial fibrillation; RAGE, receptor of advanced glycation end-products; sRAGE, soluble RAGE.

There are many OS biomarkers in AF pathogenesis. However, this article summarizes the role of the OS biomarkers 8-hydroxydeoxyguanosine (8-OHdG), glutathione peroxidase (GPx), advanced glycation end-products (AGEs), superoxide dismutase (SOD), malondialdehyde (MDA), isoprostanes (IsoPs), derivatives of reactive oxidative metabolites (DROMs) that oxidize reduced glutathione (E_h_ GSH) and cysteine (E_h_ CySH), and advanced oxidation protein products (AOPPs) in the pathogenesis of AF, as explained in **Figures 1–4** and **Table 1**. To perform this literature review, multiple databases such as Google Scholar, PubMed, and ScienceDirect were searched. The search process was concluded on August 5, 2025. Various keywords, including “atrial fibrillation,” “oxidative stress,” “biomarkers,” and “pathogenesis,” were employed. It should be noted that the clinical investigations were limited to articles published in English. While the focus was on more recent studies, no specific time limit was set.

**Table 1:** Summary of Key Findings of Oxidative Stress Biomarkers in AF

Study	Year of Publication	Oxidative Stress Biomarker(s)	Key Findings in AF
Böhm et al.^13^	2016	AGEs and TBARS	AF burden was assessed 9 months following PVI. Post-procedural AGE levels showed a significant negative correlation with AF burden at both 3 and 9 months. Similarly, TBARS concentrations were significantly inversely correlated with AF burden at 9 months. AGEs and TBARS are potential predictors for AF burden after PVI.
Li et al.^15^	2010	F2-isoprostanes	Remarkably, neither the general AF nor any of its subtypes showed elevated levels of urine F2-isoprostanes, a sensitive indicator of systemic oxidative stress in vivo.
Li et al.^21^	2021	8-OHdG	Serum 8-OHdG may serve as a valuable biomarker for AF staging and for predicting both AF recurrence and the onset of POAF following therapeutic interventions.
Toyama et al.^22^	2013	8-OHdG	The study revealed significantly elevated 8-OHdG/creatinine levels in the AF group compared to the sinus rhythm group. Multivariate regression analysis confirmed that AF was an independent determinant of increased 8-OHdG/creatinine levels, after adjusting for other oxidative stress-related factors.
Peng et al.^23^	2023	8-OHdG	Elevated 8-OHdG levels may serve as a biomarker for advanced left atrial fibrosis in AF patients and implicate DNA methylation as a key genetic mechanism underlying oxidative DNA damage in the pathophysiology of AF.
Pastori et al.^24^	2016	GPx3	Diminished GPx3 levels reflect a reduced antioxidant capacity that independently elevates cardiovascular risk in AF patients, and age-related reductions in GPx3 may contribute to the heightened cardiovascular vulnerability observed in the elderly population.
Berdaweel et al.^25^	2022	GPx4	*GPX4* genetic variants may serve as potential risk factors or predictive markers for POAF.
Tanguy et al.^26^	1998	GPx	Enhancing endogenous antioxidant capacity via selenium-induced GPx upregulation confers protection against arrhythmias induced by ischemia–reperfusion.
Bezna et al.^27^	2022	GPx	Role of GPx as a potential biomarker for oxidative stress explored in young patients with non-structural cardiac arrhythmias. The reduction in GPx activity appears to contribute to arrhythmogenic electrochemical disturbances and oxidative lipid damage, independent of dyslipidemia. This lipid peroxidation may further promote the formation of anti-lipid antibodies and subclinical endothelial injury, suggesting an early predisposition to atherogenesis.
Bezna et al.^27^	2022	SOD	SOD deficiency illustrates how oxidative stress plays a role in the pathophysiology of cardiac arrhythmias, with excess oxygen radicals interfering with several pathways linked to the initiation of arrhythmias. Estimation of SOD serves as a biomarker, where reduced levels or deficiency indicate the presence of oxidative stress, thereby underscoring its involvement in the pathogenesis of cardiac arrhythmias in young individuals.
Raposeiras-Roubín et al.^29^	2012	AGEs and sRAGE	Regardless of diabetes mellitus, individuals with AF had greater plasma levels of AGEs and sRAGE, which were also positively linked to atrial dimensions, suggesting that the AGE–RAGE axis plays a part in the arrhythmogenic structural atrial remodeling.
Yamagishi et al.^30^	2019	AGE–RAGE	It examined the pathogenic significance of the AGE–RAGE axis in AF and its thromboembolic sequelae after discussing the link between type 1 diabetes and chronic hyperglycemia and the risk of AF.
Lancefield et al.^32^	2016	sRAGE and esRAGE	It has been suggested that the pathophysiological mechanism behind the development of AF involves upregulation of RAGE. The occurrence of persistent AF is predicted by both sRAGE and esRAGE, and sRAGE is higher in Caucasian AF patients.
Zheng et al.^33^	2022	AGEs, RAGE	In conclusion, by triggering the p16/Rb pathway, AGEs accelerated cellular senescence and atrial electrical remodeling, which in turn enhanced the vulnerability to AF. RAGE or the p16/Rb pathway inhibition might be a possible treatment target for AF in diabetics.
Dudley et al.^34^	2005	Superoxide (O_2_^−^)	AF caused the LA and LAA to produce more O_2_^−^. Enhanced xanthine oxidase and NAD(P)H oxidase activity helped to explain the observed rise in LAA O_2_^−^ generation. This rise in O_2_^−^ and its reactive metabolites might be a factor in the pathogenic effects of AF, including tissue remodeling, inflammation, and thrombosis.
Liu et al.^35^	2022	MnSOD	The pathophysiology of AF is caused by oxidative stress, which may be more important in paroxysmal AF than in chronic AF. The study revealed an independent correlation between the incidence of paroxysmal AF and elevated levels of circulating plasma MnSOD.
Rubanenko et al.^36^	2017	SOD	However, patients who develop post-surgical AF demonstrate paradoxically elevated SOD activity, possibly reflecting a compensatory antioxidant response to increased oxidative stress in this subgroup.
Negreva et al.^37^	2014	SOD and catalase activity	Paroxysmal AF was associated with a marked increase in SOD and catalase activity during the initial hours of clinical onset, followed by a gradual decline as sinus rhythm was restored. These findings indicate that alterations in oxidative status are strongly linked to the disorder and are likely integral to the mechanisms underlying its initiation and progression.
Intihar et al.^39^	2025	MDA	The pathophysiology of POAF has been linked to oxidative stress, and MDA is a lipid peroxidation marker, which has been suggested as a possible biomarker. Serum MDA concentrations showed no significant association with the development of POAF, indicating that lipid peroxidation by itself may not represent a key mechanism in POAF pathogenesis. These results question the utility of MDA as a reliable biomarker for POAF risk prediction.
Syafrita et al.^40^	2022	MDA	Also, compared to AF patients without cognitive impairment, those with cognitive impairment had significantly lower levels of Aβ42 and greater levels of MDA.
Mołek et al.^43^	2022	8-isoprostane	Elevated 8-isoprostane levels, partly mediated by modifications in fibrin clot structure, are linked to thromboembolic events in patients with AF, even in the presence of anticoagulant therapy.
Pignatelli et al.^44^	2015	8-iso-PGF2α and NOX2	The potential prognostic value of urine F2-IsoP and NOX2 in AF is not well established. This study sought to determine whether urine prostaglandin PGF2α (8-iso-PGF2α) and NOX2, indicators of systemic oxidative stress, may predict CV events and death in individuals with anticoagulated non-valvular AF. Levels of 8-iso-PGF2α and NOX2 levels are predictive of CV events and overall mortality in AF patients. F2-IsoP may be used in conjunction with traditional risk variables to predict CV occurrences.
Wu et al.^45^	2015	F_2_-isoprostanes and isofurans	Urine F_2_-isoprostanes and isofurans at the end of surgery were 20% and 50% higher in subjects who developed POAF.
Tahhan et al.^46^	2017	E_h_ GSH	A 10% rise in the E_h_ GSH value was associated with a 30% greater likelihood of prevalent AF, increasing to 90% when the median value was applied as a cut-off. E_h_ GSH concentrations above the median demonstrated stronger predictive value for chronic AF compared to paroxysmal AF. Furthermore, each 10% elevation in the E_h_ GSH value was linked to a 40% greater risk of developing incident AF.
Neuman et al.^47^	2007	E_h_ GSH, E_h_ CySH, and DROMs	The increase in the odds ratios for AF for E_h_ GSH, E_h_ CySH, and DROMs was 6.1, 13.6, and 15.9, respectively. There was a stronger correlation between E_h_ GSH and E_h_ CySH than between E_h_ GSH and DROMs.

*Abbreviations:* 8-OHdG, 8-hydroxydeoxyguanosine; AF, atrial fibrillation; AGEs, advanced glycation end-products; CV, cardiovascular; DROMs, derivatives of reactive oxidative metabolites; E_h_ CySH, oxidized to reduced cysteine; E_h_ GSH, oxidized to reduced glutathione; esRAGE, endogenous secretory RAGE; GPx, glutathione peroxidase; GPx3, glutathione peroxidase 3; GPx4, glutathione peroxidase 4; LA, left atrium; LAA, left atrial appendage; MDA, malondialdehyde; MnSOD, manganese superoxide dismutase; POAF, postoperative AF; PVI, pulmonary vein isolation; RAGE, receptor of advanced glycation end-products; SOD, superoxide dismutase; sRAGE, soluble RAGE; TBARS, thiobarbituric acid reactive substances.

### Role of different oxidative stress biomarkers in atrial fibrillation pathogenesis

AF is a common arrhythmia with a complex and multifactorial pathogenesis. Among the key mechanisms, OS, which is an imbalance between ROS production and antioxidant defense, plays a crucial role. OS contributes to both electrical and structural remodeling of the atria, facilitating AF onset and progression. OS is a key factor in the pathophysiology of AF. ROS cause the creation of an AF substrate through a variety of processes, which aid in the onset and maintenance of AF. There are several ways in which OS encourages atrial remodeling.^11^ Several biomarkers of OS have been implicated in this process. However, this article summarizes the role of different OS biomarkers, such as 8-OHdG, GPx, AGEs, SOD, MDA, IsoPs, DROMs like E_h_ GSH and E_h_ CySH, and AOPPs in the AF pathogenesis.

The prevention or treatment of AF is then covered, along with a number of medicinal approaches that address OS. Enhancing the timing, targeting, and minimizing off-target effects of therapeutic strategies targeting oxidative injury in AF is imperative, given the intricate biology of ROS-induced remodeling, the evolution of ROS sources, and the compartmentalization that occurs during the progression of AF.^11^

The development of AF is significantly influenced by structural and electrical alterations. While the exact pathophysiology of paroxysmal AF (PAF) is yet unknown, DNA damage and OS seem to be significant causes. Until recently, no research had examined the connections between PAF, DNA damage, and total oxidant status (TOS). Then, in a 2021 study, TOS and DNA damage in PAF patients were investigated.^12^ In this study, the oxidative stress index (OSI), total antioxidant capacity, and TOS were used to evaluate OS. DNA damage was measured using 8-OHdG. Regarding baseline characteristics, there were no notable variations between the groups. High-sensitivity C-reactive protein, TOS, OSI, and 8-OHdG levels were substantially higher in PAF patients as compared to the control group. The sole independent predictor of PAF, according to multivariate logistic regression analysis, was the serum TOS level. PAF was predicted by TOS ≥ 12.2 with 82% sensitivity and 76% specificity. Compared to the control group, it was found that patients with PAF had considerably higher levels of TOS and DNA damage. Therefore, individuals at an increased risk of AF can be identified by TOS and DNA damage.

According to the current paradigm, OS and AF are related. Böhm et al.^13^ sought to determine whether concentrations of OS indicators before and after pulmonary vein isolation (PVI) were related to the percentage of time spent in AF. Fructosamine, AOPPs, thiobarbituric acid reactive substances (TBARS), and AGEs were tested in plasma, and the AF load was noted both before and 3 months after PVI. The concentration of post-procedural AGEs was substantially inversely associated with AF burden at 3 months (ρ = −0.63; *P* < .01) and 9 months (ρ = −0.5; *P* = .04), respectively. Additionally, the concentration of TBARS was considerably inversely associated with AF burden at 9 months (ρ = −0.61; *P* = .01). AGEs and TBARS were found to be potential predictors of AF load following the PVI. The production of fibrotic tissue increases with the amount of OS following PVI. This results in improved pulmonary vein electrical isolation and, ultimately, a reduced burden of AF.

Angina, arrhythmia, myocardial damage, and cardiac dysfunction are the hallmarks of the two most prevalent clinical cardiac diseases, AF and ischemic heart disease (IHD), which greatly increase cardiovascular (CV) morbidity and mortality and place a significant socioeconomic burden on society globally. The majority of current therapies for these two diseases are ineffective and only address symptoms.^14^ Therefore, the development of new treatments based on the underlying pathophysiological processes is urgently needed. Oxidative DNA damage may be a key underlying process that contributes to several heart disorders, including AF and IHD. As it has been demonstrated that antioxidants, nicotinamide adenine dinucleotide (NAD^+^) boosters, and enzymes involved in oxidative DNA repair processes reduce oxidative DNA damage, they may be used as therapeutic targets for AF and IHD. Hu et al.^14^ provided an overview of the primary molecular mechanisms underlying oxidative DNA damage and repair in both nuclei and mitochondria. Also, they described how oxidative DNA damage contributes to the development of AF and IHD, and lastly explored possible targets for therapeutic approaches based on oxidative DNA repair for these two cardiac conditions.

Another study supports a strong link between inflammation and arrhythmia by demonstrating that inflammatory biomarkers were considerably elevated in AF patients. Remarkably, neither the general AF nor any of its subtypes showed elevated levels of urine F2-IsoPs, a sensitive indicator of systemic OS in vivo.^15^

The oxidase system may be a major modulator of atrial OS that results in the development of AF following heart surgery, as atrial NADPH oxidase activity is independently linked to an elevated risk of postoperative AF (POAF).^16^

There is growing recognition that AF is mostly caused by epicardial adipose tissue (EAT). Apart from adipocytes’ direct infiltration into the heart, which alters conduction characteristics, EAT may also encourage an arrhythmogenic substrate through endocrine and paracrine actions. It has been demonstrated that EAT predominantly produces oxidase-dependent ROS in contrast to subcutaneous fat. Although myocardial ROS is known to have a part in the development of AF, EAT OS has not been thoroughly studied. Atrial electrical reorganization and the onset of AF are facilitated by OS in the EAT. Previously, the impact of NADPH oxidase 2 (NOX2) short hairpin RNA (shRNA)-based EAT-restricted gene therapy on atrial electrical remodeling in the short-term canine atrial tachypacing (ATP) model of AF was assessed.^17^ The ATP model involved the insertion of a single-chamber pacemaker. The atria were removed from the animals when they had persistent AF for 4–6 weeks. The controls were unpaced animals. Separately, a group of rats had an open-chest gene-injection operation limited to the EAT (using a scrambled sequence or a plasmid expressing NOX2 shRNA) before starting ATP for 9 days. AF inducibility and the regional atrial effective refractory period (ERP) were assessed by a terminal electrophysiological investigation. Compared to unpaced controls, animals with ATP-induced AF had substantially higher levels of NOX2 expression in their EAT. In addition to the atrial myocardium, DNA oxidative damage was widely distributed throughout the EAT, according to immunohistochemistry for 8-OHdG. AF inducibility following short-term ATP was reduced in animals that received NOX2 shRNA-expressing plasmids in the EAT compared to controls. An ERP-shortening attenuation was linked to this. Crucially, the left atrial (LA) appendage (LAA), which is EAT-free, also showed a similar impact in addition to the posterior LA. NOX2 expression is elevated in the canine ATP model of AF EAT. The impact of EAT OS on the development of an atrial arrhythmogenic substrate is consistent with the worldwide attenuation of LA electrical remodeling caused by EAT-restricted NOX2 inhibition.

The purpose of another study was to determine whether n-3 fatty acid and antioxidant vitamin supplementation might strengthen the antioxidant system and lower the risk of POAF.^18^ The effectiveness of treatment for preventing POAF is still lacking. The pathophysiology of cardiac arrhythmia is mostly caused by OS, while antioxidant reinforcement has yielded conflicting findings. Immediately following surgery, indicators of OS and inflammation were elevated in placebo patients; these levels were significantly reduced by antioxidant treatment. SOD, GPx, and catalase activity in supplemented patients’ atrial tissue were 17.1%, 19.7%, and 24.0% greater than the corresponding placebo values. In addition, the NADPH oxidase p47-phox subunit protein and mRNA expression in the atrial tissue of individuals who had AF were 38.4% and 35.7% greater, respectively, than in patients in sinus rhythm (SR). POAF was positively impacted by this low-cost, safe, and well-tolerated regimen that included n-3 polyunsaturated fatty acid supplementation along with vitamins C and E. It also enhanced antioxidant capability and reduced inflammation and OS.

It has been proposed that the pathophysiology of AF involves OS. OS and AF are indeed more common as people age. Nevertheless, it is unclear how the redox state and AF are related. In one study, the authors found^18^ a connection between OS and abnormal intracellular Ca^2+^ release through type 2 ryanodine receptor (RyR2), which in turn causes AF.^19^ In contrast to those in SR, they demonstrated that the atria of patients with persistent AF have oxidized RyR2. Two mouse models with intracellular Ca^2+^ leak caused by RyR2 mutations were used to analyze the molecular mechanism relating RyR2 oxidation to AF. Increased atrial RyR2 oxidation, mitochondrial dysfunction, ROS generation, and AF vulnerability were all seen in mice with intracellular Ca^2+^ leak. Both pharmacological therapy of RyR2 leakage and genetic reduction of mitochondrial ROS generation prevented AF. According to these findings taken together, changes in RyR2 and the production of ROS in the mitochondria create a vicious loop that contributes to the development of AF. It would be helpful to focus on this yet-unknown mechanism to develop efficient treatments and prevention strategies for AF. **Table 1** provides a comprehensive summary of the key findings regarding OS biomarkers in AF.

### 8-Hydroxydeoxyguanosine

It has been demonstrated that patients with OS-associated diseases had higher levels of 8-OHdG in their serum or urine; this was one of the successful discoveries of the late 1980s. 8-OHdG, a well-known biomarker for oxidative DNA damage, illustrates how ROS affect cellular DNA.^20^

Recent investigations have highlighted the pivotal role of oxidative DNA damage in the pathogenesis of AF. In this context, the circulating oxidative DNA damage marker, 8-OHdG, has emerged as a potential biomarker for assessing AF stage and identifying individuals at risk for AF recurrence and POAF. Li et al.^21^ aimed to evaluate the association between serum 8-OHdG levels and AF progression, recurrence following therapeutic intervention, and the development of POAF after cardiac surgery. Serum 8-OHdG concentrations were quantified using enzyme-linked immunosorbent assay (ELISA) in samples obtained from control individuals without a history of AF, patients with PAF or persistent AF undergoing electrical cardioversion or PVI, and patients in SR undergoing cardiac surgery. AF recurrence was assessed over a 12-month follow-up period. This study demonstrated a progressive and statistically significant elevation in 8-OHdG levels correlating with AF severity. Furthermore, patients experiencing AF recurrence post-PVI exhibited significantly higher 8-OHdG levels compared to those without recurrence. Similarly, among SR patients undergoing cardiac surgery, elevated 8-OHdG concentrations were observed in individuals who developed POAF relative to those who did not. These findings suggest that serum 8-OHdG may serve as a valuable biomarker for AF staging and for predicting both AF recurrence and the onset of POAF following therapeutic interventions.

OS is increasingly recognized as a contributing factor in the pathophysiology of AF. Toyama et al.^22^ evaluated OS levels in AF patients and assessed changes following the restoration of SR. OS was quantified using urinary concentrations of 8-OHdG, a marker of oxidative DNA damage, and biopyrrin, an oxidative metabolite of bilirubin. In study 1, urinary 8-OHdG/creatinine ratios were compared between patients with permanent AF (AF group, n = 40) and those in SR (SR group, n = 133). In study 2, changes in 8-OHdG and biopyrrin levels were assessed in 36 patients with persistent AF who achieved SR through either electrical/pharmacological cardioversion or radiofrequency catheter ablation. Analysis revealed significantly elevated 8-OHdG/creatinine levels in the AF group compared to the SR group. Multivariate regression analysis confirmed that AF was an independent determinant of increased 8-OHdG/creatinine levels, after adjusting for other OS-related factors. In study 2, all patients maintained SR during the chronic phase, and both 8-OHdG/creatinine and biopyrrin/creatinine levels were significantly reduced compared to baseline values. OS is markedly elevated in AF patients but may be attenuated with successful SR restoration. The data further suggest a self-perpetuating mechanism by which AF exacerbates its progression through the induction of OS.

Previous research has established a link between the oxidative DNA damage marker 8-OHdG and the onset or recurrence of AF. A two-part study aimed to further elucidate this relationship by investigating the association between 8-OHdG levels and the extent of LA fibrosis quantified by voltage mapping (part I) and identifying genetic factors regulating 8-OHdG levels in AF patients (part II).^23^ Before catheter ablation, plasma 8-OHdG levels were measured, and genomic DNA was extracted for genotyping. LA voltage mapping was conducted during SR, and patients were stratified into four fibrosis stages based on the percentage of low-voltage area (LVA): stage I (<5%), stage II (5%–10%), stage III (10%–20%), and stage IV (>20%). In part I (n = 209), plasma 8-OHdG levels progressively increased with advancing LVA stages, indicating a strong association between oxidative DNA damage and the severity of atrial fibrosis (stage I, 8.1 [6.1–10.5] ng/mL; stage II, 8.5 [5.7–14.1] ng/mL; stage III, 14.3 [12.1–16.5] ng/mL; stage IV, 13.9 [10.5–16.0] ng/mL; *P* < .000). In part II (n = 175), a gene-set enrichment analysis of genome-wide association study data revealed that the gene set associated with “DNA methylation on cytosine” was significantly correlated with plasma 8-OHdG levels. These findings suggest that elevated 8-OHdG levels may serve as a biomarker for advanced LA fibrosis in AF patients and implicate DNA methylation as a key genetic mechanism underlying oxidative DNA damage in the pathophysiology of AF.

### Glutathione peroxidase

GPx is indeed a valuable OS biomarker, primarily due to its role in detoxifying harmful ROS such as hydrogen peroxide and lipid hydroperoxides. GPx uses glutathione to reduce these ROS, thus preventing cellular damage. When OS increases, the demand for GPx activity rises, making it a useful indicator of the body’s antioxidant defense status. Glutathione peroxidase 3 (GPx3) is an antioxidant enzyme responsible for degrading hydrogen peroxide that plays a protective role against thrombosis. However, its involvement in CV pathology remains insufficiently characterized. Pastori et al.^24^ investigated the prognostic value of GPx3 and other antioxidant enzymes in 909 patients with AF, with a mean follow-up duration of 43.4 months. Baseline serum levels of GPx3, SOD, catalase, and markers of OS, including serum NOX2 and urinary 11-dehydro-thromboxane B2, were measured. Over the follow-up period, 160 CV events were recorded, corresponding to an annual incidence of 4.9%. Patients who experienced CV events exhibited significantly lower baseline levels of GPx3 (*P* < .001) and SOD (*P* = .037) compared to those without events. The Kaplan–Meier survival analysis revealed that patients with GPx3 and SOD activities below the median had significantly reduced survival probabilities (*P* < .001 and *P* = .010, respectively). Following multivariate Cox regression analysis, GPx3 emerged as the sole independent antioxidant predictor of CV events (hazard ratio, 0.647; 95% confidence interval [CI], 0.524–0.798; *P* < .001). Furthermore, GPx3 activity demonstrated significant inverse correlations with urinary 11-dehydro-thromboxane B2 (β = −0.337; *P* < .001) and serum NOX2 (β = −0.423; *P* < .001), indicating its role in modulating oxidative and thrombotic pathways. Notably, GPx3 activity declined progressively with advancing age, particularly among individuals aged ≥70 years. These findings suggest that diminished GPx3 levels reflect a reduced antioxidant capacity that independently elevates CV risk in AF patients, and that age-related reductions in GPx3 may contribute to the heightened CV vulnerability observed in the elderly population.

The variable incidence of POAF following cardiac surgery suggests that genetic and physiological factors may predispose certain individuals to increased risk. Glutathione peroxidase 4 (GPx4), a critical selenoenzyme that detoxifies lipid peroxides, is a key mediator of OS implicated in arrhythmogenesis that may play a role in this susceptibility. Berdaweel et al.^25^ aimed to investigate whether single-nucleotide variants within the *GPX4* gene are associated with POAF and whether these genetic variants correlate with alterations in GPx4 protein content or enzymatic activity in atrial myocardial tissue. To this end, sequencing of the *GPX4* coding region on chromosome 19 was conducted in a cohort of 189 patients undergoing elective coronary artery bypass graft (CABG) surgery, with or without valve repair. Atrial myocardial tissue from the same patients was analyzed to quantify GPx4 protein levels and enzymatic activity. POAF occurred in 25% of the cohort. Five *GPX4* variants showed significant associations with POAF risk (permutation-adjusted *P* ≤ .05), while eight variants were linked to altered myocardial GPx4 content or activity (*P* < .05). Notably, rs713041, one of the identified variants, is a recognized genetic modifier of CVD risk. These findings suggest that *GPX4* genetic variants may serve as potential risk factors or predictive markers for POAF. Moreover, the observed genotype–phenotype correlations provide mechanistic insights into how genetic variation in *GPX4* may influence enzyme function and postoperative arrhythmic outcomes, warranting further investigation in future studies.

Oxidative radicals have been implicated as key contributors to the development of reperfusion arrhythmias (RAs). However, previous attempts to mitigate RAs using various exogenous oxyradical scavengers have produced inconsistent results. One study aimed to evaluate whether enhancing endogenous antioxidant defense specifically through upregulation of GPx, a key enzyme involved in the detoxification of peroxides within cardiac tissue, could reduce the incidence and severity of RA in isolated heart preparations.^26^ To achieve this, male Wistar rats (n = 15) were fed a selenium (Se)-enriched diet (1.5 mg Se/kg) for 10 weeks to boost GPx activity, while a control group (n = 15) received a standard diet containing 0.05 mg Se/kg. The occurrence of early ventricular arrhythmias was then assessed during the reperfusion period following 10 min of regional ischemia induced ex vivo via the left coronary artery ligation. Notably, Se supplementation significantly elevated the animals’ systemic Se status and resulted in a marked reduction in RA severity, as reflected by lower arrhythmia scores. Further, the incidence of ventricular tachycardia decreased significantly (91% in controls vs. 36% in the Se group; *P* < .05), and irreversible ventricular fibrillation was completely prevented in the Se group (45% in controls vs. 0%; *P* < .05). These protective effects were accompanied by a significant increase in both mitochondrial and cytosolic GPx activity in the left and right ventricles. Overall, these findings support the hypothesis that enhancing endogenous antioxidant capacity via Se-induced GPx upregulation confers protection against ischemia–reperfusion-induced arrhythmias. The results further suggest that peroxides play a central role in the pathogenesis of reperfusion injury and may represent a therapeutic target in its prevention.

GPx is a key antioxidant enzyme and plays a crucial role in modulating cellular OS, which is implicated in the pathogenesis of various diseases. Another study investigated variations in GPx activity among patients with arrhythmic, non-structural cardiac disorders.^27^ For analysis, a total of 120 participants with a mean age of 33 years were enrolled and divided into three equal groups: two patient groups with cardiac arrhythmias, one with comorbid dyslipidemia and one without, and a healthy control group. GPx activity was measured using an enzymatic assay based on the oxidation of GSH by cumene hydroperoxide catalyzed by GPx. The findings demonstrated a significant reduction in GPx activity among arrhythmic patients, both with and without dyslipidemia, showing mean activity levels at 66% and 74% of control values, respectively (*P* < .05), indicating a state of OS. Subgroup analysis revealed GPx deficiencies ranging from 18%–35% across various arrhythmic conditions, including 29%–35% in sinus bradycardia, 31%–35% in complex arrhythmias, 30%–33% in sinus tachycardia, 27%–33% in AF, 32%–33% in atrial flutter, 27%–32% in atrial extrasystoles, 28%–30% in ventricular extrasystoles, and 18%–26% in paroxysmal supraventricular tachycardia. The most pronounced deficiency was observed in sinus bradycardia, while the least was seen in paroxysmal supraventricular tachycardia. Overall, the study results support the role of GPx as a potential biomarker for OS in young patients with non-structural cardiac arrhythmias. The reduction in GPx activity appears to contribute to arrhythmogenic electrochemical disturbances and oxidative lipid damage, independent of dyslipidemia. This lipid peroxidation may further promote the formation of anti-lipid antibodies and subclinical endothelial injury, suggesting an early predisposition to atherogenesis. Therefore, assessing GPx levels could help identify OS in arrhythmic patients and guide antioxidant-based interventions aimed at preventing the progression to atherogenic vascular dysfunction.

### Advanced glycation end-products

AGEs can be used as OS biomarkers because their formation is accelerated by OS and they, in turn, contribute to further oxidative damage. AGEs are formed through non-enzymatic glycation, where sugars such as glucose react with proteins and lipids, and this process is exacerbated by OS. The interaction of AGEs with their receptor (RAGE) further amplifies OS, creating a vicious cycle. In the pathophysiology of diabetes and its related consequences, AGEs are a wide range of compounds produced by non-enzymatic glycosylation, and RAGE is essential. According to recent research, the AGE–RAGE axis may hasten the development of CV disorders, such as arrhythmia, pulmonary hypertension, myocarditis, atherosclerosis, heart failure, and other associated disorders.^28^ Whatever its role in diabetes, the AGE–RAGE axis plays a complex role in the development and progression of CVDs. These processes include inflammation, autophagy flux changes, OS, and mitochondrial malfunction. On the contrary, the AGE–RAGE axis may be successfully disrupted by preventing AGE synthesis, preventing RAGE from attaching to its ligands, or suppressing RAGE expression, which would postpone or improve the aforementioned disorders. Both AGE and the soluble receptor for AGEs (sRAGE) may be new indicators of heart disease.

Recent research has revealed that inflammation and OS may facilitate the interaction between AGEs and RAGE. The pathophysiology of AF may be influenced by these mechanisms; however, their role is unclear. Another study investigated the relationship between AF and the AGE–RAGE axis in both diabetic and non-diabetic individuals, as the axis seems to be important in the process.^29^ In this transversal investigation, 97 consecutive outpatients were enrolled. A total of 38 patients had persistent AF, while 49 patients were in SR. Measurements of sRAGE and plasma fluorescent AGEs were made, and patients with and without AF were compared. The AF group had higher levels of fluorescent AGEs and sRAGE (74.9 ± 25.6 vs. 61.8 ± 20.1 a.u. for fluorescent AGEs, *P* = .006; 1714.2 ± 1105.5 vs. 996.1 ± 820.7 pg/mL for sRAGE, *P* = .001). These distinctions were particularly noticeable in people without diabetes. For fluorescent AGEs and sRAGE, respectively, there was a direct correlation between the two variables and LA dimensions (*r* = 0.496; *r* = 0.536 for atrial area and *r* = 0.491; *r* = 0.511 for atrial volume; *P* < .001). Fluorescent AGEs and sRAGE were found to be indicators of AF in a multivariate study, regardless of diabetes, LA distension, and other confounding factors. Regardless of diabetes mellitus, individuals with AF had higher plasma levels of AGEs and sRAGE, which were also positively linked to atrial dimensions, suggesting that the AGE–RAGE axis plays a part in the arrhythmogenic structural atrial remodeling.

There is growing evidence that people with diabetes, particularly those with poor glycemic control or long-term diabetes, have a greater risk of AF. AGEs may arise and accumulate as a result of the increased non-enzymatic glycation of amino acids in proteins, lipids, and nucleic acids that occurs with normal aging and/or diabetes. By interacting with the cell surface RAGE, AGEs not only change the tertiary structure and physiological function of macromolecules but also trigger fibrotic and inflammatory responses, which is how they contribute to aging-related diseases. One study examined the pathogenic significance of the AGE–RAGE axis in AF and its thromboembolic sequelae after discussing the link between type 1 diabetes and chronic hyperglycemia and the risk of AF.^30^

AGEs cause a pro-inflammatory phenotype when they are present in the heart. Another study examined the unknown existence of AGEs in the atrial tissue of individuals with AF.^31^ A total of 33 AF patients and 9 controls had their LAA tissue examined for the presence of the main AGEs N^ε^-(carboxymethyl)lysine (CML), vascular cell adhesion protein 1 (VCAM-1), neutrophilic granulocytes, lymphocytes, and macrophages in the myocardium and fat tissue independently. An analysis was also performed on the overall amount of fibrosis. AF patients had a considerably greater prevalence of CML in the LAA blood vessels than controls, regardless of diabetes mellitus. VCAM-1 expression in blood vessels and the quantity of infiltrating neutrophilic granulocytes, lymphocytes, and macrophages were considerably higher in AF patients than in controls, with the largest concentrations occurring in the adipose tissue; fibrosis was not statistically different between the two groups. It is interesting to note that the overall quantity of CML and fibrosis in AF and control patients showed a favorable correlation. Lastly, there was no difference in the presence of CML, VCAM-1 expression, inflammatory cells, and fibrosis among AF patients according to the AF type or surgical reason. It showed that, in AF, the intramyocardial blood vessels of the LAA have a higher pro-inflammatory condition and CML presence, which corresponds to a localized rise in inflammatory cell counts.

It has been suggested that the pathophysiological mechanism behind the development of AF involves upregulation of RAGE. Another study aimed to determine whether sRAGE levels are linked to AF in Caucasian patients.^32^ Serum levels of sRAGE and endogenous secretory RAGE (esRAGE) were assessed in 587 prospectively recruited patients. Of these patients, 527 had SR, 32 had chronic AF (length >7 days, n = 32), and 28 had PAF (duration <7 days, n = 28). Compared to individuals with SR, those with AF were older and more likely to have heart failure. Patients with persistent AF had higher circulating RAGE levels (median sRAGE, 1190 [724–2041] pg/mL and median esRAGE, 452 [288–932] pg/mL) than those with SR (sRAGE, 782 [576–1039] pg/mL and esRAGE, 289 [192–412] pg/mL; *P* < .001) or PAF (sRAGE, 799 [583–1033] pg/mL and esRAGE, 279 [201–433] pg/mL; *P* ≤ .01). Multivariable logistic regression analysis revealed that sRAGE (odds ratio [OR], 1.1 per 100 pg/mL; 95% CI, 1.0–1.1; *P* = .001), esRAGE (OR, 1.3 per 100 pg/mL; 95% CI, 1.1–1.4; *P* < .001), age, and heart failure were independent predictors of persistent AF. The only two factors that independently predicted PAF were age and heart failure. When compared to PAF, sRAGE (OR, 1.1 per 100 pg/mL; 95% CI, 1.1–1.2; *P* = .007) and esRAGE (OR, 1.3 per 100 pg/mL; 95% CI, 1.0–1.5; *P* = .017) independently predicted persistent AF in AF patients. The occurrence of persistent AF is predicted by both sRAGE and esRAGE, and sRAGE is higher in Caucasian AF patients.

Diabetes mellitus is a prevalent long-term metabolic disorder resulting from a substantial buildup of AGEs. Heart problems are a typical side effect of diabetes mellitus. The function and mechanism of atrial myocyte aging in the vulnerability to AF in diabetes were investigated. Rapid transesophageal atrial pacing was employed to track the mice’s vulnerability to AF. Mouse atrial myocytes and a single HL-1 cell were used to record the ion channels and action potential (AP) using a whole-cell patch clamp. In the same study, the link between diabetes, aging, and AF was more significantly examined using RAGE small-interfering RNA (siRNA) AAV9 and anti-RAGE antibodies.^33^ The study findings demonstrated that, in diabetic mice, greater vulnerability to AF can be linked to heightened levels of p16 and retinoblastoma (Rb) protein in the atrium. Mechanistically, AGEs increased p16/Rb protein expression and the number of senescence-associated β-galactosidase–positive cells; prolonged the AP duration; and reduced protein levels of Cav1.2 and Kv1.5 and the current density of *I_Ca,L_* and *I_Kur_* in HL-1 cells. In vitro and in vivo, these effects were reversed by anti-RAGE antibody and RAGE-siRNA AAV9 application, respectively. Moreover, AGE-mediated downregulation of Cav1.2 and Kv1.5 protein expression was inhibited by siRNA downregulating p16 or Rb. In conclusion, by triggering the p16/Rb pathway, AGEs accelerated cellular senescence and atrial electrical remodeling, which in turn enhanced the vulnerability to AF. RAGE or p16/Rb pathway inhibition might be a possible treatment target for AF in diabetics.

### Superoxide dismutase

SOD can be a useful biomarker of OS, with levels potentially increasing or decreasing depending on the cellular response to OS. SOD’s role as a first-line defense against ROS means that changes in its activity can indicate the body’s attempt to counteract oxidative damage. Numerous etiopathogenic variables, including OS, are involved in cardiac arrhythmias, which are often identified in young individuals. Another study’s goal was to assess how different forms of SOD, an antioxidant enzyme that has a physiological function in converting highly reactive oxygen free radicals into oxygen and water, affect young patients who have cardiac arrhythmias.^27^ When comparing patients to controls, it was found that mean SOD values had decreased by 61%. All arrhythmia types showed decreasing variation: AF (n = 51, 54%), sinus bradycardia (n = 54, 86%), atrial flutter (n = 55, 71%), extrasystolic ventricular arrhythmia (n = 64, 20%), extrasystolic atrial arrhythmia (n = 65, 27%), combined arrhythmias (n = 65, 93%), supraventricular paroxysmal tachycardia (n = 71, 32%), and sinus tachycardia (n = 74, 24%). SOD deficiency illustrates how OS plays a role in the pathophysiology of cardiac arrhythmias, with excess oxygen radicals interfering with several pathways linked to the initiation of arrhythmias. Those with weak antioxidant nutrition and females (n = 60, 57%) had a greater SOD decline than men (n = 67, 60%). The estimation of SOD serves as a biomarker, where reduced levels or deficiency indicate the presence of OS, thereby underscoring its involvement in the pathogenesis of cardiac arrhythmias in young individuals. This also suggests its potential utility for monitoring and therapeutic modulation through both pharmacological and non-pharmacological interventions.

The risk of stroke is elevated in AF nearly solely because of emboli from LAA thrombi. It has been recently found that AF is linked to endocardial dysfunction, which showed up as an increased expression of plasminogen activator inhibitor-1 and a decreased generation of NO in the LA and LAA. The elevated generation of superoxide (O_2_^−^) may be linked to the decreased LAA NO levels seen in AF. In one study, electron spin resonance and SOD-inhibitable cytochrome C reduction assays were used to assess the amount of O_2_^−^ produced from acutely separated heart tissue during a week of AF in pigs caused by fast atrial pacing.^34^ In comparison to control animals with similar ventricular heart rates, the LA and LAA showed 2.7-fold (*P* < .01) and 3.0-fold (*P* < .02) increases in basal O_2_^−^ production, respectively. The cytochrome C reduction test revealed a comparable three-fold (*P* < .01) increase in LAA O_2_^−^ production. Differences in atrial total SOD activity were unable to account for the increases. Either apocynin or oxypurinol decreased LAA O_2_^−^, suggesting that both xanthine oxidases and NADPH had a role in the enhanced O_2_^−^ production in AF. Increases in the activity of xanthine oxidase and LAA NAD(P)H oxidase were verified by enzyme tests of atrial tissue homogenates. While the expression of the NADPH oxidase subunits remained unchanged, the generation of superoxide increased along with GTP-loaded Rac1, an NADPH oxidase activator. AF caused the LA and LAA to produce more O_2_^−^. Enhanced xanthine oxidase and NAD(P)H oxidase activity helped to explain the observed rise in LAA O_2_^−^ generation. This rise in O_2_^−^ and its reactive metabolites might be a factor in the pathogenic effects of AF, including tissue remodeling, inflammation, and thrombosis.

Evidence suggesting that OS and inflammation have a role in the pathophysiology of AF is mounting. Manganese superoxide dismutase (MnSOD) has not yet been thoroughly studied in the development and maintenance of AF. Investigating the potential correlation between plasma MnSOD levels and AF was the goal of one study.^35^ Following screening, it included 130 consecutive patients with AF as the case group (87 had PAF, 43 had chronic AF) and 58 patients without a history of AF as the control group. Laboratory, echocardiogram, and baseline clinical data were gathered. Nicotinamide adenine dinucleotide phosphate oxidase 4 and MnSOD plasma levels were assessed using the ELISA technique. The study employed multivariable logistic regression analysis to identify the independent determinants of AF. MnSOD levels were lowest in the controls, highest in the PAF group, and lowest in the chronic AF group. The levels in the PAF group were considerably higher than those in the controls, but there was no discernible difference between the persistent AF group and the controls or between the PAF group and the persistent AF group. Notably, the MnSOD levels in the case group showed a positive association with the red cell distribution width coefficient of variation (*r* = 0.214; *P* = .014) and a significantly negative correlation with left atrial diameter (LAD) (*r* = −0.232; *P* = .008), according to Spearman correlation analysis. The best cut-off value of MnSOD in predicting PAF, as determined by receiver operating characteristic (ROC) curve analysis, was 311.49 μg/mL (sensitivity, 52.9%; specificity, 77.6%; area under the curve, 0.668). Multivariate logistic regression analysis revealed that MnSOD levels (OR, 1.003; 95% CI, 1.001–1.005; *P* = .002) were an independent risk factor for PAF. The pathophysiology of AF is caused by OS, which may be more important in PAF than in chronic AF. The study also revealed an independent correlation between the incidence of PAF and elevated levels of circulating plasma MnSOD.

To assess SOD levels in patients with coronary heart disease (CHD) undergoing CABG and to evaluate its potential role in the development of post-surgical AF (PSAF), a total of 96 CHD patients scheduled for CABG were enrolled.^36^ The cohort was divided into two groups: group 1 included patients without PSAF, and group 2 included patients who developed new-onset AF in the early postoperative period. Compared to group 1, SOD levels were significantly elevated in group 2 (2589.8 ± 1999.3 vs. 1572.8 ± 1275.2 U/g; *P* = .034). Patients with PSAF were older on average (64.0 ± 8.4 vs. 57.9 ± 7.3 years; *P* = .048) and had a longer history of CVD (86.9 ± 76.1 vs. 44.3 ± 38.4 months; *P* = .002). Additionally, PSAF patients more frequently exhibited class III angina (72.4% vs. 47.8%; *P* = .028), class III heart failure (38.0% vs. 7.5%; *P* = .006), and had significantly larger LADs (43.5 ± 4.1 vs. 37.9 ± 3.4 mm; *P* < .001). Multivariate analysis identified two independent predictors of PSAF: an LAD of >41 mm (OR, 5.1; 95% CI, 2.1–9.8; *P* = .0005) and a SOD level of >2948 U/g (OR, 4.4; 95% CI, 1.1–8.9; *P* = .04). CABG is associated with increased OS, which is generally accompanied by reduced SOD levels due to enzymatic consumption. However, patients who develop PSAF demonstrate paradoxically elevated SOD activity, possibly reflecting a compensatory antioxidant response to heightened OS in this subgroup.

The processes linked to AF’s clinical presentation are of significant interest to researchers. The activity of the antioxidant enzymes catalase and SOD in individuals experiencing PAF (duration <48 h) was dynamically assessed.^37^ The characteristics under investigation were analyzed in the erythrocytes of 51 patients (59.84 ± 1.60; 26 men) at the time of admission, 24 h later, and 28 days after SR was restored. There were also 52 controls (59.50 ± 1.46; 26 men) who had no prior history of arrhythmia. To treat the irregular heartbeat, propafenone was administered. Spectrophotometry was used to measure the enzyme activity. From 2 to 24 h, the average duration of AF events until admission was 8.14 h. While the patient was in the hospital, their SOD and catalase activity was significantly greater than that in the controls. Also, the activity was markedly elevated in PAF, even in the early hours of the disorder’s clinical presentation, but gradually diminished when the SR returned. Oxidative status alterations are intimately linked to the disease and likely contribute to the mechanisms behind its onset and clinical progression.

### Malondialdehyde

The peroxidation of polyunsaturated fatty acids produces MDA in vivo. In addition to its potential for atherogenicity, MDA interacts with proteins. MDA creates lysine–lysine cross-links when it reacts with lysine residues.^38^ MDA is a well-established biomarker for assessing OS, a condition where there is an imbalance between the production of ROS (free radicals) and the body’s ability to counteract them. MDA is a byproduct of lipid peroxidation, a process where free radicals damage cell membranes and other lipid-containing structures. Elevated levels of MDA are often found in various diseases and conditions associated with increased OS, making it a useful indicator for the diagnosis and monitoring of treatment effectiveness.^39^

A frequent side effect of heart surgery is POAF, which is linked to higher morbidity and longer hospital stays. The pathophysiology of POAF has been linked to OS, and MDA is a lipid peroxidation marker, which has been suggested as a possible biomarker. There are, however, conflicting data about its predictive usefulness. The relationship between serum MDA levels and the incidence of POAF in patients following heart surgery has been investigated in a prospective observational analysis, where 99 consecutive patients undergoing elective on-pump heart surgery were enrolled. Patients requiring dialysis for chronic renal disease, emergent surgery, or prior AF were not included. Preoperatively; intraoperatively; following aortic clamp release; and at 8, 24, 48, and 72 h postoperatively were the six perioperative periods at which blood samples were obtained for MDA measurement.^39^ Of the patients, 33 (33%) had POAF. Significantly older patients (*P* = .017) and those with higher EuroSCORE II scores (*P* = .019) were those who developed POAF. The blood MDA concentrations of POAF and non-POAF patients did not differ significantly at any time point. POAF was more common in individuals having valvular surgery (*P* = .014). As serum MDA levels were not linked to the development of POAF, it is possible that lipid peroxidation is not the primary cause of POAF pathogenesis. The predictive efficacy of MDA for POAF risk categorization is called into question by these findings. In order to avoid POAF, future studies should investigate other OS indicators and their possible therapeutic applications.^39^

Also, compared to AF patients without cognitive impairment, those with cognitive impairment had significantly lower levels of Aβ42 and greater levels of MDA.^40^

### Isoprostanes

The polyunsaturated fatty acid arachidonic acid, which is found in the phospholipids of cell membranes, is peroxidized to produce stable, prostaglandin-like molecules known as IsoPs.^41^ The cyclooxygenase enzyme, which catalyzes the synthesis of prostaglandins from arachidonic acid, is not necessary for the creation of IsoPs from arachidonic acid.^42^

IsoPs are reliable biomarkers for measuring OS in vivo, particularly in situations where other markers might be unreliable. They are formed by the non-enzymatic peroxidation of arachidonic acid, a process triggered by free radicals. Elevated levels of IsoPs indicate increased lipid peroxidation and, consequently, heightened OS. There is increased OS in AF patients; however, it is uncertain how this affects the safety and effectiveness of anticoagulant medication. Another study aimed to determine whether clinical outcomes in individuals with anticoagulated AF are related to 8-isoprostaglandin F2 (8-isoprostane) levels.^43^ Serum 8-isoprostane was examined in 243 AF patients (median age, 69 years), as well as prothrombotic indicators such as von Willebrand factor (VWF), fibrinolytic proteins, endogenous thrombin potential (ETP), plasma fibrin clot permeability, and clot lysis time (CLT). Major bleeding, mortality, and ischemic cerebrovascular episodes were documented throughout a median follow-up of 53 months while using anticoagulation—primarily non-vitamin K antagonist oral anticoagulants. Patients with arterial hypertension, PAF, or chronic AF and women all had elevated 8-isoprostane levels. Further, higher fibrinogen, lower VWF, and higher plasminogen activator inhibitor 1 levels were observed in patients with 8-isoprostane levels of ≥559 pg/mL (the top quartile) compared to those with 8-isoprostane levels of <250 pg/mL (the bottom quartile). Additionally, there was no difference in CHA_2_DS_2_-VASc score, CLT, or ETP. Patients with thromboembolic events exhibited concentrations of 8-isoprostane that were 48.6% greater than those of the other patients. At follow-up, thromboembolic events were linked to 8-isoprostane levels of >459 pg/mL, which was the best cut-off value (hazard ratio, 2.87; 95% CI, 1.17–7.03; *P* = .02). Neither significant bleeding (2.0%/year) nor all-cause mortality (1.9%/year) was linked to 8-isoprostane. To sum up, elevated 8-isoprostane levels, partly mediated by modifications in fibrin clot structure, are linked to thromboembolic events in patients with AF, even in the presence of anticoagulant therapy.

The potential prognostic value of urine F2-IsoP and NOX2 in AF is not well established. One study sought to determine whether urine prostaglandin PGF2α (8-iso-PGF2α) and NOX2, indicators of systemic OS, may predict CV events and death in individuals with anticoagulated non-valvular AF. ^44^ In this prospective analysis, 1002 patients with anticoagulated AF were monitored for a median of 25.7 months. All significant CV events, CV deaths, and all-cause fatalities were regarded as the study’s main outcomes. Myocardial infarction, transient ischemic attack, cardiac revascularization, and fatal or non-fatal ischemic stroke were among the CV events. OS indicators were examined, including serum sNOX2-dp, a marker of NOX2 activation, and urine 8-iso-PGF2α. Thirty-one non-CV deaths and 78 CV deaths were recorded among the 125 patients who experienced a CV episode. A correlation was found between 8-iso-PGF2α and sNOX2-dp (*R*_s_ = 0.765; *P* < .001). 8-iso-PGF2α and sNOX2-dp were shown to have a significantly greater cumulative incidence of CV events and CV fatalities across tertiles. Across urine 8-iso-PGF2α tertiles, an elevated risk of all-cause mortality was seen. CV and non-CV fatalities, as well as CV occurrences, were predicted by 8-iso-PGF2α in Cox or Fine and Gray models. When 8-iso-PGF2α tertiles were added to the CHA_2_DS_2_-VASc score, the ROC curves for each outcome and the nutritional risk index for CV events improved (0.24 [0.06–0.53]; *P* = .0067). Levels of 8-iso-PGF2α and NOX2 are predictive of CV events and overall mortality in AF patients, according to the study. F2-IsoP may be used in conjunction with traditional risk variables to predict CV occurrences.

POAF is mostly caused by OS, according to research on animals. However, it is unclear how much OS indicators may be associated with human POAF risk. In 551 patients undergoing heart surgery, the correlation between incident POAF and validated fatty acid–derived OS indicators (F_2_-IsoPs, isofurans, and F_3_-IsoPs) in plasma and urine was evaluated. At enrollment, the conclusion of surgery, and on the second postoperative day, biomarkers were assessed. Centrally adjudicated, POAF lasting ≥30 s was verified by electrocardiography or a rhythm strip. Results were evaluated until the patient was released from the hospital or until the 10^th^ postoperative day, whichever came first. After surgery, the levels of each OS indicator in urine increased (2–3 times higher than baseline, *P* < .001), but, by postoperative day 2, they had decreased to levels similar to the baseline.^45^

Plasma concentrations, on the contrary, were mostly constant over the perioperative period. After surgery, the levels of urine F_2_-IsoPs and isofurans were 20% and 50% greater in those who experienced POAF. Although there was no significant correlation between baseline biomarker levels and POAF, IsoPs and isofurans showed reasonably linear relationships with POAF after surgery and on the second postoperative day. For instance, urine isofurans and F_3_-IsoPs had extreme quartile multivariate adjusted ORs (95% CI) of 1.95 (1.05–3.62; *P* for trend = .01) and 2.10 (1.04–2.25; *P* for trend = .04) after surgery, respectively. There was minimal variation in the correlations of biomarkers with POAF by medication usage, surgery type, and demographics (*P* ≥ .29 for each). These new findings contribute to the growing body of data suggesting that enhanced OS has a major pathogenic role in POAF.^45^

### Derivatives of reactive oxidative metabolites that oxidize reduced glutathione and cysteine

In experimental research, OS may be a major cause behind the onset of AF; however, there is currently little information on this in humans. Glutathione, cysteine, and their oxidized derivatives are examples of aminothiols whose levels in the blood may be measured to determine systemic OS. One study investigated whether glutathione (E_h_ GSH) and cysteine redox potentials might be linked to incident and prevalent AF. A total of 1439 individuals who had undergone coronary angiography had their plasma levels of aminothiols tested; 148 (10.3%) of these patients were diagnosed with AF. A total of 104 out of 917 patients (11.5%) experienced incident AF after a median follow-up of 6.3 years. Multivariate logistic regression and Cox regression models were used to determine whether OS markers were independent predictors of prevalent and incident AF after adjustment for traditional risk factors, heart failure, coronary artery disease, and high-sensitivity C-reactive protein level. For each 10% increase in E_h_ GSH, the odds of prevalent AF were 30% higher (OR, 1.3; 95% CI, 1.1–1.7; *P* = .02) and 90% higher (OR, 1.9; 95% CI, 1.3–2.7; *P* = .004) when the median was used as a cut-off. An E_h_ GSH value above the median was more predictive of chronic AF (OR, 4.0; 95% CI, 1.3–12.9; *P* = .01) than of PAF (OR, 1.7; 95% CI, 1.1–2.7; *P* = .03). Each 10% increase in the E_h_ GSH value was associated with a 40% increase in the risk of incident AF (hazard ratio, 1.4; 95% CI, 1.1–1.7; *P* = .01). A higher OS as determined by glutathione redox potentials is linked to both incident and prevalent AF. It is necessary to research therapies that alter OS to cure and prevent AF.^46^

Antioxidant medications have shown anti-arrhythmic benefits in humans, and AF has been linked to myocardial OS. In people with and without AF, blood indicators of oxidation and related inflammation were examined. Forty male participants with or without persistent or permanent AF were matched for age, sex, diabetes, and smoking status, known confounding factors for OS measurement, in a cross-sectional, case–control study to compare serum markers of inflammation and OS. This study used DROMs and ratios of E_h_ GSH and E_h_ CySH to quantify OS, and it measured inflammatory markers, including high-sensitivity C-reactive protein, interleukins-1β and -6, and tumor necrosis factor-α. Univariate, conditional logistic regression analysis revealed that OS, but not inflammatory markers, was statistically significantly associated with AF (*P* < .05). The increase in the ORs for AF for E_h_ GSH, E_h_ CySH, and DROMs was 6.1 (95% CI, 1.3–28.3; *P* = .02), 13.6 (95% CI, 2.5–74.1; *P* = .01), and 15.9 (95% CI, 1.7–153.9; *P* = .02), respectively. There was a stronger correlation between E_h_ GSH and E_h_ CySH (*r* = 0.66) than between E_h_ GSH and DROMs (*r* = 0.41). AF and OS were still significantly associated in multivariate analysis that adjusted for statins and other AF risk variables that varied between the groups. These findings imply that OS indicators can be useful in predicting how to treat AF.^47^

### Advanced oxidation protein products

AOPPs are a growing number of useful biomarkers for evaluating OS. They are created when ROS, especially hypochlorous acid, oxidize proteins. AOPPs may be more accurate indicators of total OS as they are thought to be more persistent than other oxidative damage indicators, such as lipid peroxidation products. AOPPs are a risk factor for several CV conditions on their own.^48^

One significant mechanism in cardiac remodeling in chronic kidney disease (CKD) has been linked to cardiomyocyte apoptosis. AOPPs’ impact on cardiomyocyte apoptosis and the ensuing cardiac remodeling in CKD is yet unclear, though. The function of AOPPs in cardiomyocyte apoptosis in CKD has been evaluated. The cardiomyoblast cells of H9C2 rats were subjected to AOPPs. One study investigated the expression of endoplasmic reticulum stress, JNK signaling, and apoptotic markers (cleaved caspase-3 and Bax). AOPPs markedly accelerated the death of H9C2 rat cardiomyoblast cells in vitro via activating c-Jun N-terminal kinase (JNK) signaling and endoplasmic reticulum stress. Through OS inhibition, apocynin somewhat mitigated these effects. Serum AOPP levels in CKD rats were significantly higher than those in sham-operated animals during the duration of the study (*P* < .05).^48^ Further, cardiomyocyte apoptosis and serum AOPP levels were strongly correlated (*R*^2^ = 0.76; *P* < .01). To sum up, AOPPs exacerbated cardiomyocyte apoptosis in vitro, whereas apocynin partially counteracted these effects by inhibiting JNK signaling and endoplasmic reticulum stress through OS inhibition. However, it remains unknown whether the elevated AOPPs in the CKD model in vivo contribute to the pathophysiology of the heart. In order to enhance cardiac remodeling in CKD, these findings imply that pharmaceutical strategies to reduce AOPP-aggravated cardiomyocyte apoptosis may be helpful.^48^

OS biomarkers provide insight into the underlying redox imbalance contributing to AF. They reflect oxidative damage to lipids, proteins, and DNA, as well as compromised antioxidant defenses. Monitoring these biomarkers may aid in risk stratification, early detection, and potentially targeted antioxidant therapy in AF. New biomarkers that represent pathophysiologically significant oxidative signaling cascades in important cellular microdomains within the CV system may eventually replace the biomarkers that are now in use. However, in order for this to happen, the redox biology community and clinical researchers must collaborate to conduct studies that do more than merely confirm that a signal is associated with the seriousness of disease. The focus must be on evaluating the marker’s predictive power over conventional clinical measurements and its potential to be used to customize treatment for each patient and enhance results.^49^

## Conclusion

OS plays a critical role in the pathogenesis of AF by promoting structural and electrical remodeling of the atria. This article concludes that OS biomarkers, including 8-OHdG, GPx, AGEs, SOD, MDA, IsoPs, DROMs, E_h_ GSH, and E_h_ CySH, play a significant role in the pathogenesis of AF, as explained in **Figures 1–4**. Future research is required on advanced oxidation protein products in AF pathogenesis. Clinically, these biomarkers offer potential for early detection, risk stratification, and monitoring of therapeutic response in AF patients, supporting their integration into predictive and personalized medicine strategies.
